# Injection tip method to improve poor visibility during over-the-scope clip procedure

**DOI:** 10.1055/a-2423-0284

**Published:** 2024-10-25

**Authors:** Koichiro Kawano, Mamoru Takenaka, Reiko Kawano, Daisuke Kagoshige, Koutaro Mine, Katsuhisa Nishi, Masatoshi Kudo

**Affiliations:** 1 13865 Gastroenterology, Hyogo Prefectural Awaji Medical Center, Sumoto, Japan; 2 38158 Department of Gastroenterology and Hepatology, Kindai University Faculty of Medicine Graduate School of Medical Sciences, Osakasayama, Japan

The over-the-scope (OTS) clip is a widely used endoscopic device for hemostasis and fistula closure 1 2 3 4 . During endoscopic procedures, extraneous matter, such as blood clots or fecal masses, often becomes lodged in the hood on the tip of the endoscope, obstructing the view. Flushing with the waterjet nozzle is often ineffective owing to the small volume of water and the distance between the waterjet nozzle and the forceps hole. Therefore, a syringe is commonly used to flush water through the forceps insertion hole to clear obstructions ( Fig. 1 ). However, when the OTS clip system is attached to the endoscope, the handwheel structure restricts the size of syringe that can be used, and the small, rigid forceps insertion hole does not permit the use of a regular syringe ( Fig. 2 ). To address this issue, we developed a syringe “injection tip” for easier flushing during OTS clipping procedures.

**Fig. 1:**
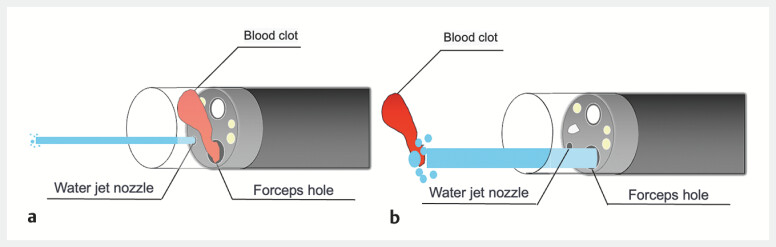
**a** As the blood clots are aspirated, they become lodged in the forceps hole and obstruct the field of view. Only a small volume of water is pumped through the waterjet outlet, and the distance between the forceps hole and the waterjet outlet is relatively large, so the blood clots stuck in the forceps hole are not removed. **b** Pumping water through the forceps hole provides a larger volume, effectively removing the blood clots stuck there.

**Fig. 2:**
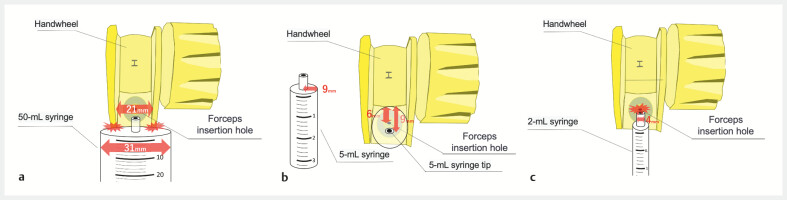
**a** The 50-mL syringe (31-mm diameter) cannot fit over the forceps insertion hole because the width of the handwheel is only 21 mm around the forceps insertion hole. This interferes with operating the handwheel. **b** Although the 5-mL (18-mm diameter) syringe is thinner than the width of the wheel, the distance from the wheel to the forceps insertion hole is only 6 mm. Therefore, the tip of the syringe (radius 9 mm) cannot reach the hole. **c** A 2-mL (10-mm diameter) syringe can be placed over the forceps insertion hole. However, the over-the-scope (OTS) clip forceps insertion hole is narrower and tighter than a standard one, making it difficult to insert a regular syringe with a 4-mm outer diameter tip.

We used a 16G fistula cannula (Covidien Japan), cut in half and with one of the cut halves attached to a 50-mL syringe ( Fig. 3 ). The thin cannula tip (1.5 mm) could easily be inserted into the OTS system forceps insertion hole ( Fig. 4 ), enabling forceful water flushing that effectively cleared blood clots and restored visibility.

**Fig. 3:**
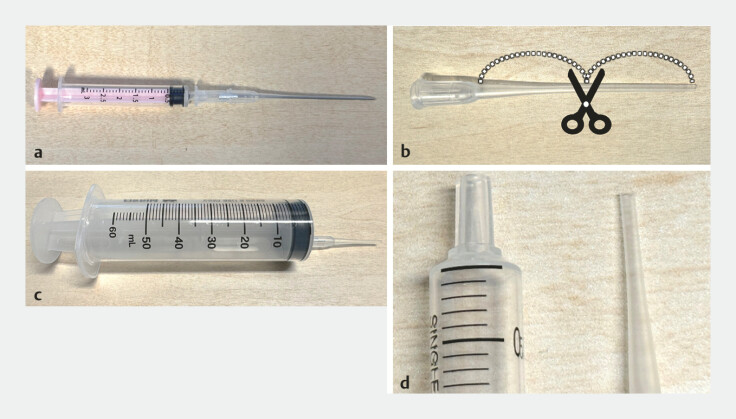
The injection tip: **a** 16G fistula cannula; **b** the needle is removed and the cannula cut in half; **c** a 50-mL syringe with one half of the cut cannula attached; **d** the syringe tip and cut cannula tip.

**Fig. 4:**
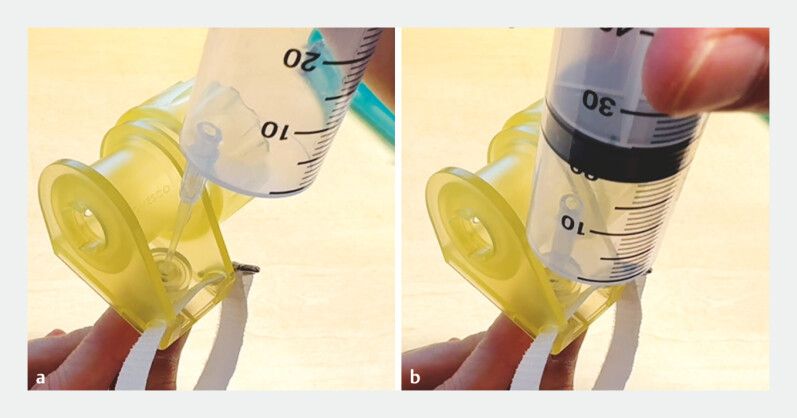
The cannula is inserted into the forceps insertion hole of the OTS clip system without any resistance.

The patient was a 60-year-old man presenting with bloody stools. Endoscopy revealed active bleeding from an erythematous transverse colonic diverticulum 5 . After an OTS clip had been attached to the endoscope tip, a blood clot obstructed the endoscopic view. We flushed the area using the injection tip method described above with a 16G fistula cannula, which improved the endoscopic view. Hemostasis was achieved with OTS clipping ( Fig. 5 , Video 1 ). The patient recovered well and was discharged on postoperative day 5.

**Fig. 5:**
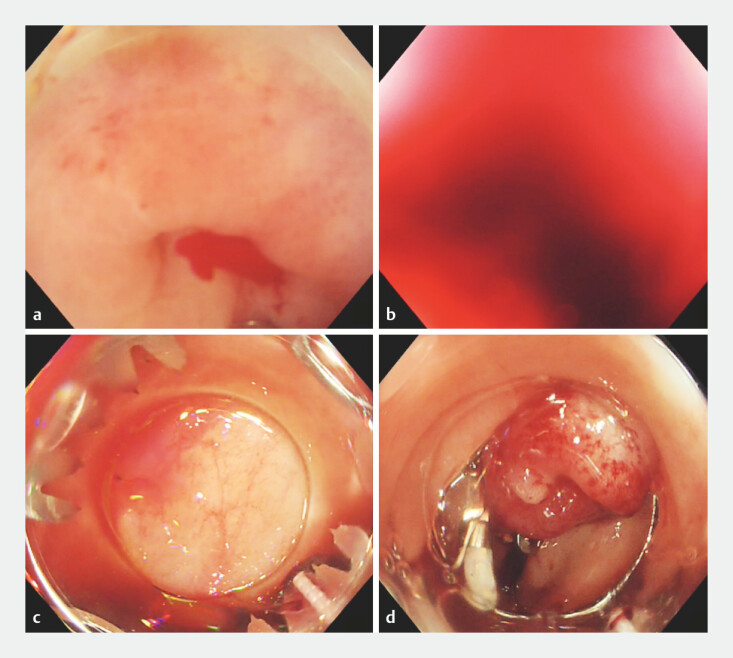
**a** Active bleeding from a transverse colon diverticulum. **b** Aspirated blood clots obscure the field of view. **c** The clots have been removed and visibility restored. **d** An OTS clip is placed in the bleeding diverticulum to achieve hemostasis.

During over-the-scope (OTS) clip procedures the endoscopic view is obscured by aspirated extraneous matter. The injection tip technique provides effective flushing through the forceps hole, enabling hemostasis and fistula closure.Video 1

We present a simple, effective injection tip method to improve poor visibility during OTS clipping procedures.

Endoscopy_UCTN_Code_TTT_1AU_2AF

## Notes

Endoscopy E-Videos https://eref.thieme.de/e-videos
*E-Videos* is an open access online section of the journal *Endoscopy* , reporting on interesting cases and new techniques in gastroenterological endoscopy. All papers include a high-quality video and are published with a Creative Commons CC-BY license. Endoscopy E-Videos qualify for HINARI discounts and waivers and eligibility is automatically checked during the submission process. We grant 100% waivers to articles whose corresponding authors are based in Group A countries and 50% waivers to those who are based in Group B countries as classified by Research4Life (see: https://www.research4life.org/access/eligibility/ ). This section has its own submission website at https://mc.manuscriptcentral.com/e-videos .
